# Integrated Evaluation of Undernutrition, Anaemia, and Intestinal Parasitic Infections in School-Aged Children: A Cross-Sectional Study in Three Regions of Southern Madagascar

**DOI:** 10.3390/children12080990

**Published:** 2025-07-28

**Authors:** Gabriela Tapia-Veloz, Mónica Gozalbo, Venny Guirao, Hafsa Dinari, Màrius Vicent Fuentes, María Trelis

**Affiliations:** 1 Parasite & Health Research Group, Department of Pharmacy, Pharmaceutical Technology and Parasitology, Faculty of Pharmacy, University of Valencia, 46100 Valencia, Spain; gabriela.tapia@uv.es (G.T.-V.); mario.v.fuentes@uv.es (M.V.F.); 2 Department of Medicine and Public Health, Science of the Food, Toxicology and Legal Medicine, University of Valencia, 46010 Valencia, Spain; monica.gozalbo@uv.es (M.G.); hafdi@alumni.uv.es (H.D.); 3 Department of Health, ONG Bel Avenir, Toliara 601, Madagascar; agentdesante@ongbelavenir.org; 4 Joint Research Unit on Endocrinology, Nutrition and Clinical Dietetics, University of Valencia-Health Research Institute La Fe, 46026 Valencia, Spain

**Keywords:** undernutrition, anaemia, soil-transmitted helminths, child, adolescents, Madagascar

## Abstract

**Background/Objectives:** Undernutrition and intestinal parasitic infections are critical public health problems in low-income countries, with adverse effects on child growth and increasing anaemia. Madagascar, with a high prevalence of these factors, lacks comprehensive studies analysing their interaction. This study aimed to assess the nutritional status, the prevalence of anaemia, and the occurrence of intestinal parasitic infections among children and adolescents in three southern regions of Madagascar. **Methods:** A cross-sectional, prospective study of 289 children and adolescents (10–18 years) from three schools located in Antsoamadiro, Fianarantsoa, and Toliara was conducted. Sociodemographic, anthropometric, and haemoglobin concentration data, as well as faecal samples, were collected. Nutritional status was assessed by Nutrimetry, combining Height-for-Age and BMI-for-Age indicators. Stool samples were analysed by optical microscopy and molecular methods. **Results:** Nutricode 1 (short stature/stunting + thinness/wasting) was significantly more frequent in Toliara. Nutricode 1 was also significantly more prevalent in males than females. Anaemia affected 57.8% of participants and was significantly associated with Nutricode 1. The overall parasitism rate was also associated with Nutricode 1. *Trichuris trichiura* and *Ascaris lumbricoides* significantly increased the risk of stunting, wasting, and Nutricode 1. Co-infection with *Trichuris trichiura* + *Giardia duodenalis* was significantly associated with wasting and Nutricode 1. This co-infection was also related to the presence of anaemia, as was moderate-intensity infection with *T. trichiura*. **Conclusions:** There is a high co-burden of undernutrition, anaemia, and parasitic infections in southern Madagascar. These findings highlight the urgency of implementing comprehensive health programmes combining parasite control, nutritional support, and iron supplementation adapted to regional realities.

## 1. Introduction

The prevalence of child undernutrition, manifested mainly as stunting and wasting, is concentrated in regions such as sub-Saharan Africa, South Asia, and the Caribbean. Undernutrition, in particular, represents a significant public health concern in sub-Saharan Africa, where stunting rates range from 20.2% to 48.1%, and underweight prevalence varies between 14% and 36.5% [1,2]. The aetiology of undernutrition is multifactorial, encompassing economic, social, and political determinants [3].

Madagascar exhibits one of the highest poverty rates globally, with over 90% of the population living on less than USD 3.10 per day. This situation of economic vulnerability is exacerbated by extreme climatic conditions—such as prolonged droughts, cyclones, and floods—particularly in the southern regions of the country, resulting in severe food insecurity [4]. Additionally, limited access to safe drinking water and sanitation services further compounds public health challenges, especially among children [5,6,7]. The country also exhibits alarming levels of hunger, ranking 124th out of 127 countries in the 2024 Global Hunger Index. Approximately 39.7% of the Malagasy population is undernourished, with 39.8% of children under five experiencing chronic undernutrition (stunting), 7.2% suffering from acute undernutrition (wasting)—with higher rates reported in the south (9.2%) and southeast (9.4%)—and an under-five mortality rate of 6.6% [4,8]. Collectively, these factors contribute to a high burden of undernutrition among Malagasy children and adolescents.

Anaemia represents another critical health determinant that significantly affects the Malagasy population, particularly among age groups with elevated nutritional requirements and limited iron reserves. The prevalence of anaemia in Madagascar is high, affecting 46% of children and 26% of women [4]. The highest regional data reported was in Toliara (southwest) at 76%, while the lowest was in the capital of the country, Antananarivo (north), at 64% [9]. The causes of anaemia are multifactorial and include inadequate or insufficient intake of essential nutrients, such as iron, vitamin B12, folate, vitamin A, and phosphate; hereditary conditions; and chronic diseases [10,11]. Several studies have highlighted the association between iron-deficiency anaemia (IDA) and undernutrition, with stunting identified as a potential risk factor for the development of IDA [12]. An often-under-recognised contributor to anaemia is infection with pathogenic organisms, which can lead to a chronic state [10].

Intestinal parasitic infections (IPIs) constitute a further significant public health risk, particularly in populations living in poverty and without adequate access to WASH services (water, sanitation, and hygiene) [13]. In Madagascar, the prevalence of soil-transmitted helminths (STHs) ranges from 0% to 94%, with endemic transmission reported in 111 out of the 114 districts [14]. Schistosomiasis is also highly prevalent and endemic in 107 districts, with reported prevalence rates reaching up to 89% [14,15,16]. Regarding protozoa with an impact on child development, previous epidemiological studies have reported *Giardia duodenalis*, ranging from 7% to 79% of the population [13,17,18,19]. School-aged children represent the most vulnerable group to IPIs, consistently exhibiting the highest morbidity rates. Moreover, co-infection with multiple parasites of varying intensities may impact both nutritional status and anaemia levels in complex ways, with chronic intestinal infections identified as one of the most common contributing factors [20].

Multiple studies have identified a strong association between IPIs—such as STHs (e.g., *Ascaris lumbricoides* and *Trichuris trichiura*), the trematode *Schistosoma mansoni*, and the intestinal protozoan *G. duodenalis*—and childhood undernutrition. These parasites adversely affect health by impairing digestion and nutrient absorption, inducing chronic inflammation, and causing nutrient loss, all of which contribute to stunting, underweight, and wasting [21,22,23]. IPIs are also closely associated with anaemia. The mechanisms include intestinal mucosal damage that exacerbates nutrient deficiencies; chronic blood loss in the caecum and colon due to sustained injury caused by adult helminths; splenic sequestration of erythrocytes; and inflammation-mediated anaemia. High-intensity helminth infections have also been consistently linked to elevated anaemia prevalence [20,24,25].

The aim of this study was to assess the nutritional status, the prevalence of anaemia, and the occurrence of intestinal parasitic infections among school-aged children in three regions of southern Madagascar, focusing on their interrelationship and impact on child health. An integrated approach combining anthropometric methods, such as Nutrimetry, and haematological and parasitological methods was used. By analysing these parameters, the study aims to elucidate the interactions between malnutrition, anaemia, and intestinal parasitic infections, as well as to characterise the local epidemiological context in areas where limited or no data are available.

## 2. Materials and Methods

### 2.1. Study Design and Description of Study Sites

A prospective, cross-sectional observational study was conducted among 289 children and adolescents aged between 10 and 18 years. Recruitment was conducted in three schools managed by the non-governmental organisation (NGO) *Bel Avenir* (Madagascar) in collaboration with the *Agua de Coco* Foundation (Spain). The number of participants per school was determined proportionally to the total number of students enrolled in each school to ensure a representative sample. At *École des Salines*, the school with the largest student population, 159 participants were recruited, of whom 62 were girls residing in the *Foyer*, a social home for girls in vulnerable situations, also managed by *Bel Avenir*. At *École des Saphirs*, 71 students were recruited, and at *Ferme École*, 59 students.

**Figure 1:**
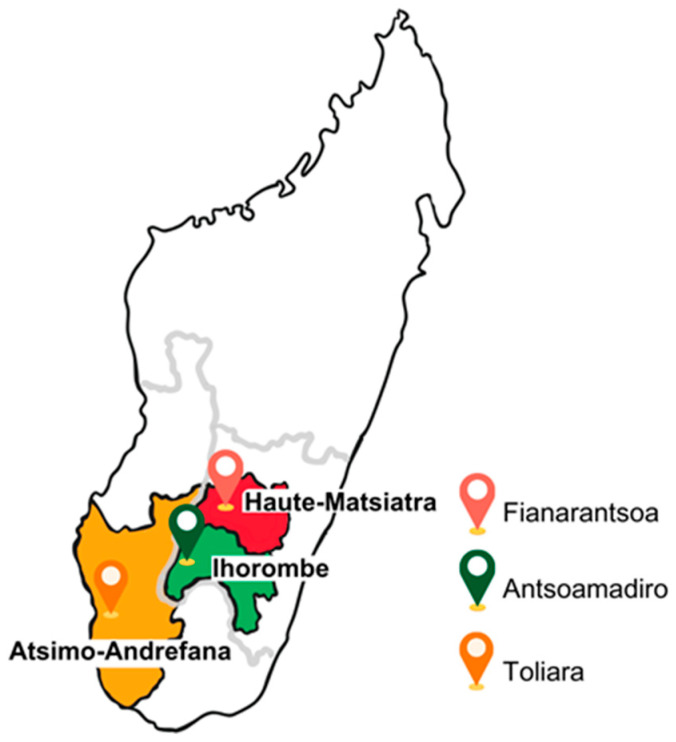
Geographical location of the three municipalities, Fianarantsoa, Antsoamadiro, and Toliara, surveyed in the Haute-Matsiatra, Ihorombe, and Atsimo-Andrefana regions.

The *École des Saphirs* is located in the village of Antsohomadiro, within the Ihorombe region; the *Ferme École* is situated in the city of Fianarantsoa, in the Haute-Matsiatra region; and both the *École des Salines* and the *Foyer* are based in the city of Toliara, in the Atsimo-Andrefana region (Figure 1). Data and sample collection were carried out in 2023, during two separate phases. The first phase, from January to March, involved fieldwork in Toliara. The second phase, conducted from June to July, encompassed the municipalities of Antsohomadiro and Fianarantsoa.

The three study locations reflect the geographical, climatic, and socio-economic diversity of southern Madagascar, thereby offering an overview of varied living conditions across regions. The Ihorombe region has a dry savannah climate, with an annual mean temperature of 23.8 °C [26]; the Haute-Matsiatra region has a temperate climate with dry winters and an average annual temperature of 19.1 °C [27]; and the Atsimo-Andrefana region is characterised by a hot, arid climate, with a mean temperature of 25.3 °C [28]. With regard to socio-economic indicators, the Multidimensional Poverty Index (MPI)—which measures deprivation across health, education, and standard of living—registers high values in Ihorombe (0.532) and Atsimo-Andrefana (0.527), while Haute-Matsiatra presents a lower MPI of 0.429. The prevalence of out-of-school population also varies markedly: 52.1% in Atsimo-Andrefana, 36.8% in Ihorombe, and 10.4% in Haute-Matsiatra [29].

At local level, Antsoamadiro (Ihorombe) is a rural area with limited access to basic services, where the economy relies heavily on small-scale sapphire mining. Fianarantsoa (Haute-Matsiatra), a more urbanised area, combines agriculture, tourism, and improved access to infrastructure and services. Toliara (Atsimo-Andrefana), a coastal city with large peri-urban zones, primarily depends on artisanal fishing and salt production. These regions are highly vulnerable to environmental stressors, including prolonged droughts and cyclone-associated flooding—pressures that have intensified due to climate change. This environmental fragility has led to a critical food security crisis, particularly acute in Atsimo-Andrefana and, to a lesser extent, in Ihorombe.

### 2.2. Recruitment and Sample

Most participants came from families with low or very low socioeconomic status. With the support of a staff member of the NGO Bel Avenir, informational visits and meetings were conducted at each establishment for the parents or legal guardians of the participants. These sessions aimed to explain the study’s objectives and procedures.

Parents or legal guardians who provided written informed consent were given a stool sampling kit, accompanied by detailed collection instructions. Faecal samples were collected during school hours or submitted the following day at the respective schools or establishments. Samples were then promptly taken to the laboratory/dispensary located at each establishment, where they were processed and stored. Each participant also underwent a structured interview to gather sociodemographic information. Nutritional status was assessed through anthropometric evaluation, using WHO indicators and the Nutrimetry methodology. Additionally, during school hours, a capillary blood sample was obtained for anaemia screening.

### 2.3. Epidemiological Questionnaire

A face-to-face interview was conducted, either with the parents/legal guardians or directly with the participants, depending on the case. During the interview, a structured questionnaire was supplied to gather basic sociodemographic information. The questionnaire included the following items: (1) date of birth, (2) sex, (3) father’s educational level, (4) mother’s educational level, (5) employment status of both parents, and (6) availability of latrines in the household.

### 2.4. Anthropometric Analysis

**Figure 2:**
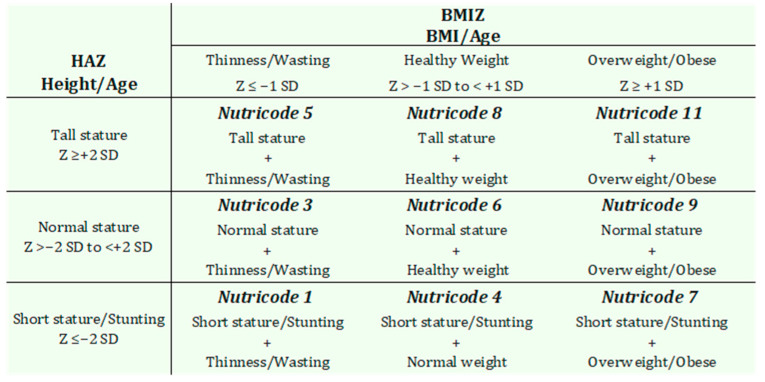
Nutricodes and their interpretations, adapted from Selem-Solís et al. [32].

Body weight and height were measured using an Omron^®^ electronic scale (Omron Healthcare Co., Ltd., Kyoto, Japan) (accuracy: 100 g) and a Seca 216^®^ mechanical stadiometer (Seca GmbH & Co. KG., Hamburg, Germany) (accuracy: 1 mm), respectively. Anthropometric measurements were conducted in accordance with the recommendations of the World Health Organization (WHO) [30]. Height-for-Age Z-scores (HAZ) and Body Mass Index-for-age Z-scores (BMIZ) were calculated using WHO AnthroPlus™ software, version 1.0.4, based on the 2007 WHO growth reference standards for children and adolescents aged 5 to 19 years [31]. These indicators enabled the classification of participants into various degrees and types of malnutrition, according to WHO reference criteria. For the final nutritional status diagnosis, the Nutrimetry approach was applied. This method integrates HAZ and Z-BMI values into a 3 × 3 matrix, allowing for a joint, visual, and more comprehensive interpretation of nutritional status [32,33]. This method applies nine Nutricodes (1, 3, 4, 5, 6, 7, 8, 9, 11), derived from the various combinations of HAZ and BMIZ (Figure 2), which are used to represent different nutritional status diagnoses [33].

### 2.5. Blood Sample Collection

Determination of capillary haemoglobin (Hb) values was performed using the portable HemoCue™ Hb 201+ (HemoCue AB, Ängelholm, Sweden) system. HemoCue microcuvettes were filled with 10 µL of capillary blood from each participant, obtained from the second and third drops of blood drawn from the middle or ring finger. The Hb concentration in the surveyed participants was classified according to the criteria established by the WHO [34]. This system has been validated in previous studies targeting both young children [35] and pregnant women [36], demonstrating that the HemoCue device provides haemoglobin measurements comparable to those obtained in laboratory settings under controlled conditions.

### 2.6. Parasitological Assessment

A total of 289 stool samples were collected, one per participant. In the field, fresh samples were processed and analysed using the Kato–Katz technique to identify and quantify intestinal helminth eggs. The parasitic load, expressed in eggs per gram of stool (epg), was estimated by multiplying the number of eggs observed on the slides by 24, based on a template capacity of 41.7 mg of stool. Infection intensity was classified according to the thresholds defined by WHO for STHs and *Schistosoma mansoni* [37,38]. Remaining faecal material was preserved in sterile tubes containing 70% ethanol and transported to the Parasitology Laboratory of the Faculty of Pharmacy, University of Valencia (Spain) for further analysis. Upon arrival in Spain, a 5 g subsample of stool from each participant was selected, concentrated, and filtered for 5 min at 2500 rpm using Midi Parasep^®^ solvent-free (SF) devices (Apacor Ltd., Wokingham, UK). The sediment obtained through centrifugation was divided into two aliquots: one used for DNA extraction and the other fixed in 10% formalin at a 1:3 ratio for microscopy analysis of the concentrate. This examination enables the identification of resistant forms of intestinal parasites (cysts and eggs).

DNA extraction was performed using the QIAamp DNA Stool Mini Kit (Qiagen, Hilden, Germany), following the manufacturer’s instructions. For the diagnosis of *G. duodenalis*, a quantitative PCR (qPCR) with high sensitivity was conducted targeting a 62-base pair (bp) fragment of the gene encoding the small subunit ribosomal RNA (SSU rRNA) of the parasite [39].

### 2.7. Statistical Analyses

A descriptive analysis of the sample was conducted using basic statistical measures, such as percentages. Subsequently, a bivariate analysis was carried out using chi-squared tests for categorical variables to identify preliminary significant associations. Binary logistic regression analyses were performed to assess the relationship between intestinal parasitic infections and variables such as nutritional status and anaemia. The strength of association was expressed as odds ratios (OR) with a 95% confidence interval (CI). A *p*-value < 0.05 was considered statistically significant. All variables were analysed using SPSS Statistics version 29.02 for Windows (IBM Corporation, Armonk, NY, USA).

## 3. Results

### 3.1. Socio-Demographic Characteristics of the Study Participants

A total of 289 children and adolescents from three schools—*École des Salines*, *École des Saphirs*, and *Ferme École*—located in the municipalities of Antsoamadiro, Fianarantsoa, and Toliara were enrolled (Table 1). The mean age of participants was 14 years, with an age range from 10 to 18 years. Females accounted for 59.9% of the sample, while males represented 40.1%. A pairwise analysis was conducted to explore associations between the study areas and various sociodemographic variables, including the educational level of the mother and father, working status of the head of household, and the presence of latrines in the household. The results showed that in Toliara, there were 3.50 times more mothers without studies compared to Fianarantsoa (95% CI: 1.82–6.74; *p* < 0.001) and 2.12 times more than in Antsoamadiro (95% CI: 1.19–3.77; *p* = 0.014). Conversely, in both Antsoamadiro (OR = 6.35; 95% CI: 1.92–21.02; *p* = 0.002) and Fianarantsoa (OR = 6.07; 95% CI: 1.76–21.03; *p* = 0.004), mothers were significantly more likely to have an elementary education compared to those in Toliara. Regarding the presence of latrines in the household, homes in Fianarantsoa were significantly more likely to have latrines compared to Toliara (OR = 8.24; 95% CI: 3.79 –17.94; *p* < 0.001) and Antsoamadiro (OR = 24.78; 95% CI: 9.77–62.83; *p* < 0.001). Additionally, the households in Toliara were 3.00 times more likely to have latrines compared to those in Antsoamadiro (95% CI: 1.52–5.93; *p* = 0.002).

### 3.2. Nutritional Status and Anaemia

Regarding nutritional status assessed through Nutrimetry (Table 2), Nutricode 1 was the most prevalent, with 32.9% of children presenting thinness/wasting accompanied by short stature/stunting. This was followed by Nutricode 4, with a prevalence of 29.8%, corresponding to individuals with a normal weight but reduced height. These findings indicate that stunting is the most common form of undernutrition in the population.

The geographical distribution of nutritional status showed significant differences in the pairwise comparisons between the study areas. School children from Toliara had a higher likelihood of presenting combined undernutrition, corresponding to Nutricode 1 (OR: 4.40; 95% CI: 0.24–5.19; *p* < 0.001), as well as a significantly greater risk of exhibiting Nutricode 3, characterised by normal size + thinness/wasting (OR: 6.486; 95% CI: 1.93–21.85; *p* < 0.001), compared with those from Fianarantsoa. In addition, children from Toliara were 5.82 times more likely to present with Nutricode 3 (95% CI: 1.10–16.96; *p* < 0.001) compared with those from Antsoamadiro. On the other hand, school children from Antsoamadiro (OR: 3.352; 95% CI: 1.73–6.51; *p* < 0.001) and Fianarantsoa (OR: 5.029; 95% CI: 2.67–9.37; *p* < 0.001) had a higher risk of short stature/stunting + normal weight (Nutricode 4) compared with those from Toliara. Finally, residents of Fianarantsoa were 4.68 times more likely (95% CI: 1.90–11.58; *p* < 0.001) to present with a normal nutritional status (Nutricode 6) compared with those from Antsoamadiro.

A stratified analysis by municipality, examining the association between sociodemographic variables (educational level of the mother and father, occupation of the head of household, and the presence of latrines in the household) and nutritional status (Nutrimetry), showed no significant associations.

When examining the distribution of Nutricodes by sex and age group, no statistically significant differences were identified (Table 3). In general terms, a similar pattern was observed between boys and girls across both age groups, although with some notable exceptions. Among boys, Nutricode 3, associated with thinness, was more prevalent in the 10–14-year-old group compared to those aged 15–18 years (16.1% vs. 7.4%). Among girls, Nutricode 1 showed a higher prevalence in the 10–14 age group compared to the older group (15.0% vs. 6.9%). The association between sex and the different Nutricodes was analysed, taking Nutricode 6 as a reference. Boys were found to be 3.62 times more likely of being classified under Nutricode 1 (95% CI: 1.78–7.40; *p* < 0.001) compared to girls. No significant associations were observed for the rest of Nutricodes.

The overall prevalence of anaemia in the study population was 57.8% (167/289). Among those affected, 28.8% (60/167) had mild levels, 33.2% (96/167) moderate, and 3.8% (11/167) severe. No statistically significant association was found between anaemia and age or sex; however, females exhibited slightly higher prevalence rates compared to males (61.3% vs. 52.6%). When examining anaemia in relation to the Nutricodes (Table 4), it was observed that children classified under Nutricode 1 were 2.20 times more likely to present with anaemia (95% CI: 1.09–4.42; *p* = 0.026) compared to those with Nutricode 6.

### 3.3. Overall Prevalence of Intestinal Parasites

The overall prevalence of IPIs in the study population was 91.0% (Table 5). Significant differences were observed in parasite prevalence between the municipalities studied, with Toliara exhibiting the highest infection rates (95.6%). Pairwise analysis between municipalities showed that schoolchildren residing in Toliara had a 3.90 times higher risk (95% CI 1.38–11.03, *p* = 0.015) of intestinal parasite infection compared to Fianarantsoa and 3.56 times higher than in Antsoamadiro (95% CI 1.30–9.79, *p* = 0.020). Similarly, the risk of *G. duodenalis* infection was significantly higher in Toliara (OR = 4.00, 95% CI 1.97–8.12, *p* < 0.001) compared to Antsoamadiro. On the other hand, children and adolescents living in Antsoamadiro were 2.83 times more at risk of T. trichiura infection compared to Fianaratsoa (OR = 2.83; 95% CI: 1.23–6.51; *p* = 0.022). School children in Fianaratsoa had a higher risk of *S. mansoni* infection (OR = 2.69, 95% CI 1.12–6.38, *p* = 0.040) compared to Antsoamadiro.

A stratified analysis by municipality examining the association between sociodemographic variables (educational level of the mother and father, occupation of the head of household, and the presence of latrines in the household) and the prevalence of parasitosis, both overall and by species, revealed no significant associations.

A single parasitic species was identified in 50.9% (147/289) of participants. Co-infections involving two species were found in 34.6% (100/289) of children, with the most common combination being *G. duodenalis* + *T. trichiura* (69/100). Additionally, 5.5% (16/289) of participants were coinfected with three or more species, most frequently *G. duodenalis* + *T. trichiura* + *A. lumbricoides*.

### 3.4. Association Between Intestinal Parasites, Nutritional Status, and Anaemia

To analyse the relationship between IPIs and nutritional status (Table 6), we first compared them with stunted children (HAZ < −2), then with those who showed thinness or wasting (BMIZ < −2), and finally with Nutricode 1, which encompasses children who show both short stature/stunting + thinness/wasting. This analysis showed that the overall rate of parasitism by *G. intestinalis*, *T. trichiura*, *A. lumbricoides*, and *S. mansoni* was associated with an increased risk of combined undernutrition, classified as Nutricode 1 (OR: 4.19; 95% CI: 1.23–14.34; *p* = 0.008). Participants with *T. trichiura* + *G. duodenalis* coinfection were 4.55 times more likely to present with thinness/wasting (95% CI: 2.45–8.47; *p* < 0.001) and 3.42 times more likely to be classified under Nutricode 1 (95% CI: 1.95–6.00; *p* < 0.001).

A significant association was also identified between the STH infections and various indicators of undernutrition (Table 6). Participants infected with geohelminths had an increased risk of stunting (OR: 3.43; 95% CI: 2.02–5.83; *p* < 0.001), thinness (OR: 4.39; 95% CI: 2.66–7.24; *p* < 0.001), and the Nutricode 1 classification (OR: 6.42; 95% CI: 3.72–11.09; *p* < 0.001). These associations remained significant when individual STH species were analysed separately. In the case of *T. trichiura*, infected individuals were 2.43 times more likely to suffer from stunting (95% CI: 1.43–4.39; *p* = 0.003), 3.56 times more likely to present with thinness (95% CI: 2.04–6.21; *p* < 0.001), and 3.45 times more likely to meet the criteria for Nutricode 1 (95% CI: 2.02–5.89; *p* < 0.001). Similarly, infection with *A. lumbricoides* was significantly associated with an increased risk of stunting (OR: 4.77; 95% CI: 2.17–10.49; *p* < 0.001), thinness (OR: 3.31; 95% CI: 1.79 –6.15; *p* < 0.001), and Nutricode 1 (OR: 6.10; 95% CI: 3.33–11.21; *p* < 0.001).

With respect to the association between intestinal parasitic infections and anaemia, participants with any intestinal parasitic infection had 2.84 times greater odds of presenting with anaemia (95% CI: 1.22–6.62; *p* = 0.013). Also, coinfection with *T. trichiura* and *G. duodenalis* was associated with the presence of anaemia (OR = 2.84; 95% CI: 1.22–6.62; *p* = 0.013). On the other hand, species-specific analysis showed that schoolchildren infected with *T. trichiura* were 2.17 times more likely to be anaemic (95% CI: 1.25–3.76; *p* = 0.004).

The intensity of infection, as determined using the Kato–Katz method for *A. lumbricoides*, *T. trichiura*, and *S. mansoni*, was compared in relation to anaemia risk. A significant correlation was observed only for *T. trichiura*, where children and adolescents with moderate infection intensity were found to be 11.98 times more likely to suffer from anaemia (95% CI: 3.594–39.983; *p* < 0.001).

## 4. Discussion

In low-income countries, such as Madagascar, undernutrition, anaemia, and intestinal parasitosis are major public health problems that particularly affect the school population. In the present study, 32.9% of the participants were classified under Nutricode 1, which corresponds to children and adolescents with short stature/stunting + thinness/wasting, followed by Nutricode 4, characterised by short stature/stunting + normal weight. These results indicate that stunting is the most prevalent form of undernutrition in the studied population. The findings of this study are consistent with previous research in other regions of the island. For example, a study conducted with children in Antananarivo reported a prevalence of 21.1% of stunting accompanied by underweight and 32.6% of chronic undernutrition [40]. Similarly, Asgary et al. identified in the Anivorano region that stunting was also the most frequent form of child undernutrition [41]. The findings are also consistent with those reported by the Global Hunger Index 2024, which identified stunting as the most frequent type of undernutrition among children in Madagascar, with a national prevalence rate of 39.8% [8].

The analysis of the municipalities studied and the Nutrimetry revealed that schoolchildren in Toliara had a higher risk of being classified in Nutricode 1 and Nutricode 3 compared to those in Fianarantsoa, as well as a higher risk of being in Nutricode 3 compared to schoolchildren in Antsoamadiro. Conversely, schoolchildren in Fianarantsoa showed better nutritional status, reflected by a higher proportion in Nutricode 6 compared to the other two municipalities. These findings indicate that schoolchildren in Toliara have the worst nutritional status. This situation could be partially related to socio-demographic factors. Comparative analysis between regions revealed that Toliara had the highest proportion of mothers with no formal education compared to Fianarantsoa and Antsoamadiro. Although this variable did not show a significant association with nutritional status in the stratified analyses by municipality, it could still represent an important vulnerability factor. Scientific evidence has consistently pointed to parental—and especially maternal—education as a key predictor of child nutritional status [41,42]. For example, a study in Indonesia found that health-promoting behaviours adopted by formally educated parents, such as vaccination and micronutrient supplementation, significantly influenced children’s nutritional status [43]. Another possible explanation is that Toliara is part of the Atsimo-Andrefana region, located in the extreme south of Madagascar, an area characterised by high levels of food insecurity. This situation is aggravated by adverse climatic events, such as prolonged droughts, as well as recurrent food crises, which severely limit the availability of and access to nutritious food [44,45]. In addition, this region has a high Multidimensional Poverty Index (0.527), which assesses deprivation in three key dimensions: health, education, and standard of living. These structural conditions reinforce the vulnerability in the nutritional status of the school population in Toliara, contributing to the results observed in this study [29].

The aetiology of undernutrition is multifactorial, and one potential influencing factor is age. In our study, Nutricode 3 was more prevalent among children aged 10 to 14 years compared to those over 15 years. Similarly, girls in the 10 to 14 age group showed a higher prevalence of Nutricode 1 compared to older adolescents. Although these associations did not reach statistical significance, the results may suggest that, within the study population, undernutrition is primarily affecting the younger age group. This stage is particularly vulnerable due to the high nutritional demands associated with growth and early pubertal development. These findings are consistent with previous studies, which observed that younger adolescents are at greater risk of undernutrition due to rapid linear growth and hormonal changes during early puberty, which increase energy and micronutrient requirements [46,47]. In particular, girls undergoing early puberty may be more susceptible to undernutrition if their dietary intake does not meet these heightened physiological needs [48].

Another factor that could contribute to undernutrition may be the sex of the child. In our study, boys were found to be 3.62 times more likely to be classified in Nutricode 1 compared to girls. These findings may be partially influenced by the specific circumstances of some of the female participants, who were living in a social shelter (*Foyer*) managed by the NGO Bel Avenir, where they received three regular meals per day. This nutritional support may have contributed to improved nutritional status of girls compared to boys, thus partially explaining the lower prevalence of undernutrition observed among the female participants in our sample. However, this pattern is consistent with findings from previous studies conducted in sub-Saharan Africa and other countries, which have also reported greater nutritional deterioration among boys [46,47,48,49,50,51]. A possible explanation relates to sociocultural factors that influence household food allocation practices, which in some contexts may lead to an unequal distribution of food portions within the household, favouring girls or boys depending on cultural norms [42]. Conversely, other studies have reported higher rates of undernutrition among girls. For example, in India, it was found that girls were more vulnerable to undernutrition due to structural factors, such as cultural preferences for boys, early marriage, and gender-biased intra-household food distribution, which exposed them to greater nutritional risk [52]. In light of these findings, it is essential to design interventions that address the root causes of undernutrition and promote equitable feeding practices for all children, regardless of their sex.

Anaemia is a medical condition with important public health implications, especially in low-income countries. In the study population, 57.8% of participants were found to be anaemic, with moderate anaemia being the most prevalent form. This figure exceeds previously reported rates in Madagascar, where anaemia prevalence among children and women was estimated at 46% and 26%, respectively [4]. Another study among children living in impoverished neighbourhoods in Antananarivo reported a prevalence of only 24.4% [10]. These discrepancies may be partly attributed to the age groups targeted in each study. Most previous research focuses exclusively on children under five years of age, whereas our sample includes older children and adolescents, who are going through puberty, a developmental stage characterised by rapid growth and increased muscular development, during which iron requirements increase substantially [11].In addition, the geographic region in which the sample was collected may play a crucial role. Some areas, such as the one in this study, face severe food insecurity, in contrast to the northern regions of Madagascar, where economic activities, such as fishing and commercial agriculture, contribute to greater food stability [44].

In relation to anaemia and sex, a higher prevalence of anaemia was observed in girls compared to boys, although the differences were not significant. This finding is consistent with previous studies that have reported a higher proportion of anaemia in females [53,54]. Sex differences in the prevalence of anaemia can be attributed to a number of factors. Biological factors include menstruation during puberty, which increases iron requirements in adolescent girls. Physiological factors also play a role, and, in some cases, social factors, like cultural norms or gender roles, may condition unequal access to food [53].

Among the potential causes of anaemia, undernutrition emerged as a significant factor. A strong correlation was observed between Nutricode 1 and the presence of anaemia, compared to Nutricode 6. This result is consistent with previous research that also documented this association [12]. One plausible explanation is that participants come from low- or very low-income households, which limits the access to a balanced diet, especially micronutrients, such as iron. The literature indicates that both undernutrition and anaemia are frequently associated with inadequate dietary practices, food insecurity, and consumption of foods of low nutritional quality [11,52]. A systematic review highlighted insufficient intake of micronutrients, particularly iron, folate, and zinc, as a recurring issue among women in low- and middle-income countries. In addition, vitamin A deficiency has also been identified as a common cause of anaemia in contexts of extreme poverty [55]. The high rates of anaemia observed in Madagascar are largely attributable to poverty and undernutrition, reflecting diets with inadequate levels of essential micronutrients, including iron, zinc, and vitamins A and B12 [19,43,56]. Given that the extreme south of the island exhibits alarming levels of food insecurity, this situation may perpetuate a vicious cycle in which food insecurity leads to undernutrition, which in turn increases the risk of anaemia [57]. Indeed, one study reported a positive association between food insecurity and the risk of anaemia, specifically iron deficiency anaemia [58].

In terms of intestinal parasitosis, the overall prevalence of IPIs in schoolchildren was 91.0%. Toliara had the highest prevalence of parasitic infections (95.6%) and a significantly higher risk of parasitism compared to Fianarantsoa and Antsoamadiro. These results highlight the poor structural and environmental conditions in Toliara. Studies in Madagascar have associated this high parasite prevalence with poverty, poor access to WASH services, and conditions that facilitate the faecal–oral cycle of transmission [19]. Previous studies have shown that improved WASH services can reduce the risk of intestinal worm infections [59]. In this context, the results obtained reinforce that Toliara is the municipality with the greatest structural vulnerabilities, so it would be necessary to implement comprehensive parasite control measures, access to safe water, improved sanitation, and hygiene education strategies, as demonstrated by successful interventions in similar settings.

Intestinal parasite infections play a key role in the aetiology of anaemia, especially in contexts of poverty. In this study, a significant association was observed between anaemia and the overall prevalence of intestinal parasitic infections, *Trichuris trichiura* + *Giardia duodenalis* co-infections, as well as individual *T. trichiura* infections. When analysing the intensity of infection using the Kato–Katz method, it was found that only moderate-intensity *T. trichiura* infections were significantly associated with anaemia. These findings are consistent with previous studies that have documented this association [60,61,62]. Chronic intestinal inflammation caused by parasitic infections, together with intestinal villous atrophy, can affect nutrient absorption and alter iron homeostasis, thereby reducing iron absorption and utilisation and suppressing haemoglobin synthesis and erythropoiesis [23]. In co-infections, the synergistic interaction between the two parasites contributes to anaemia, as reported in the studies in school populations from low-income countries [63]. Additionally, blood loss has been observed to be more significant in moderate to heavy parasitic infections. The pathogenesis of *T. trichiura* is primarily associated with mucosal damage and endogenous blood loss, which result from its burrowing mechanism into the intestinal epithelium. *T. trichiura* infections can lead to chronic bleeding in the caecum or colon, particularly at the sites where adult worms are embedded in the mucosa. It is estimated that a single *T. trichiura* worm may cause a daily blood loss of approximately 0.005 mL. Consequently, in cases of moderate to severe infections, the cumulative blood loss can become substantial, contributing significantly to the development of anaemia [19,24,62].

In many populations, intestinal parasitic infections represent a major source of nutritional stress, which has a negative impact on the growth and development of children and adolescents. In the present study, a significant association was observed between various forms of undernutrition and IPIs. Participants infected with intestinal parasites had a markedly increased risk of wasting combined with stunting (Nutricode 1). *T. trichiura* + *G. duodenalis* co-infections were significantly associated with thinness and Nutricode 1. A strong correlation was also found between the overall prevalence of STHs and stunting, as well as with Nutricode 1. These associations remained statistically significant when individual STH species—*T. trichiura* and *A. lumbricoides*—were analysed separately. The detrimental impact of IPIs, such as those examined in this study, on the nutritional status of school-aged populations is very important [22]. In addition, co-infections—both by different helminth species and by helminths and protozoa—have been identified as an aggravating factor in childhood undernutrition [22]. Studies have shown that children concurrently infected with *T. trichiura* and *A. lumbricoides*, even with low parasite loads, are at increased risk of stunting, especially when both species coexist at different levels of intensity [64]. *G. duodenalis* infection has also been associated with low weight in infancy [23]. The simultaneous presence of multiple parasitic species in the same host can exacerbate the negative effects on nutritional status by generating a synergistic interaction between pathogens that intensifies nutritional deterioration. A systematic review by Fauziah et al. revealed that stunted children are significantly more prone to STH infections compared to those with normal growth [65]. Previous research has also identified *A. lumbricoides* as the helminth most commonly associated with stunting and wasting [23,66], its pathogenic mechanisms including induction of anorexia, nutrient malabsorption, and jejunal mucosal alterations. Since *A. lumbricoides* resides in the small intestine, where digestion and nutrient absorption take place, it competes directly with the host for nutrients, thus contributing to the onset of undernutrition [66,67]. Similarly, persistent *T. trichiura* infection is associated with nutritional deficits, and the degree of damage appears to be correlated with both the duration of infection and parasite load. In particular, even after successful eradication of the parasite, growth problems, particularly in linear growth, may persist into adulthood [22,23,68]. In the case of *G. duodenalis*, infections often result in recurrent episodes of diarrhoea, compromising adequate nutrient absorption and contributing to impaired nutritional status [23]. The multiple impairments caused by parasitic infections in children and adolescents, together with additional factors such as a diet deficient in nutritional quality, create a vicious cycle: parasitic infections cause undernutrition, and malnourished children are, in turn, more susceptible to acquiring new parasitic infections.

In Madagascar, several national multisectoral programmes have been implemented to combat undernutrition, such as the National Nutrition Policy 2022–2030, the World Bank’s Multisectoral Programme ‘FAFY’, and the National School Feeding Programme, as well as mass deworming campaigns against geohelminths and schistosomiasis, with the support of the WHO. It should be noted that the NGO Bel Avenir has programmes to combat undernutrition in its schools and is also part of the national programme against geohelminthiasis. However, despite these interventions, undernutrition, anaemia, and parasitic infections remain highly prevalent in the Malagasy population. This is largely due to unresolved structural factors, such as persistent poverty, limited availability of resources, social and cultural barriers, and poor access to WASH services. Without substantial improvement in these conditions, existing programmes are unlikely to achieve a sustained and comprehensive impact on the health of the population.

## 5. Conclusions

The results of this study show a high prevalence of undernutrition, anaemia, and intestinal parasites among the schoolchildren assessed. Toliara had the highest rates of combined short stature/stunting and thinness/wasting (Nutricode 1), as well as the highest rate of parasitic infections, reflecting poor hygiene and lifestyle conditions. This positions Toliara as the most vulnerable area in this study. A higher prevalence of anaemia was observed among girls, while boys showed a higher frequency of undernutrition, underscoring the importance of designing equitable, gender-sensitive interventions that address the specific needs of both groups. It is particularly relevant to consider nutritional monitoring and potential iron supplementation for girls, without neglecting boys. Our study confirms the strong association between undernutrition, anaemia, and intestinal parasitic infections, especially those caused by *Trichuris trichiura*, *Ascaris lumbricoides*, and the co-infection of *T. trichiura* and *Giardia duodenalis*. Furthermore, it highlights the complex interaction between parasitic infections and nutritional status. These findings underline the urgent need for integrated interventions targeting children and adolescents, combining deworming programmes, improvements in dietary quality, micronutrient supplementation—particularly iron—and strategies adapted to the specific circumstances of each region.

## Figures and Tables

**Table 1:** Sociodemographic description of the population analysed according to place of residence.

Category	All	Antsoamadiro	Fianarantsoa	Toliara
	(N = 289)	(N = 71)	(N = 59)	(N = 159)
	% (n)	% (n)	% (n)	% (n)
**Sex**				
Male	40.1 (116)	46.5 (33)	49.2 (29)	34.0 (54)
Female	59.9 (173)	53.5 (38)	50.8 (30)	66.0 (105)
**Age groups in years**				
0–14	54.7 (158)	50.7 (36)	16.9 (10)	70.4 (112)
15–18	45.3 (131)	49.3 (35)	83.1 (49)	29.6 (47)
**Education level of the father**				
No studies	41.2 (119)	46.5 (33)	30.5 (18)	42.8 (68)
Elementary school	49.8 (144)	45.1 (32)	54.2 (32)	50.3 (80)
High school studies	6.6 (19)	7.0 (5)	11.9 (7)	4.4 (7)
University studies	2.4 (7)	1.4 (1)	3.4 (2)	2.5 (4)
**Education level of the mother**				
No studies	46.0 (131)	38.0 (27)	27.1 (16)	56.6 (90)
Elementary school	45.3 (133)	47.9 (34)	54.2 (32)	40.9 (65)
High school studies	7.6 (22)	14.1 (10)	13.6 (8)	2.5 (4)
University studies	1.0 (3)	0.0 (0)	5.1 (3)	0.0 (0)
**Working status of the household head (parent)**				
Yes	94.5 (273)	100.0 (71)	98.3 (58)	90.6 (144)
No	5.5 (16)	0.0 (0)	1.7 (1)	9.4 (15)
**Presence of latrine in the household**				
Yes	43.9 (127)	18.3 (13)	84.7 (50)	40.3 (64)
No	56.1 (162)	81.7 (58)	15.3 (9)	59.7 (95)

N: number of the total population analysed; %: percentage of cases; n: number of cases in each municipality.

**Table 2:** Distribution of Nutrimetry (Nutricodes) by municipality among schoolchildren in Madagascar.

Nutrimetry	All	Antsoamadiro	Fianarantsoa	Toliara
	(N = 292)	(N = 71)	(N = 59)	(N = 159)
	% (n)	% (n)	% (n)	% (n)
**Nutricode 1** (Short stature/Stunting + Thinness/wasting)	32.9 (96)	32.4 (23)	13.6 (8)	40.9 (65)
**Nutricode 3** (Normal size + Thinness/wasting)	16.4 (48)	5.6 (4)	5.1 (3)	25.8 (41)
**Nutricode 4** (Short stature/Stunting + Normal weight)	29.8 (87)	50.7 (36)	40.7 (24)	17.0 (27)
**Nutricode 6** (Normal stature + Healthy weight)	18.5 (54)	11.3 (8)	37.3 (22)	15.1 (24)
**Nutricode 7** (Short stature/Stunting + Overweight/Obese)	1.4 (4)	0.0 (0)	3.4 (2)	1.3 (2)

N: total number in each group; %: percentage of total of each group; n: number of cases in each group.

**Table 3:** Distribution of Nutrimetry (Nutricodes) by age, group, and sex among schoolchildren in Madagascar.

Nutrimetry	Age (yrs.)				Sex	
	Male		Female		Male	Female
	10–14 yrs.	15–18 yrs.	10–14 yrs.	15–18 yrs.		
	(N = 62)	(N = 54)	(N = 96)	(N = 77)	(N = 116)	(N = 173)
	% (n)	% (n)	% (n)	% (n)	% (n)	% (n)
**Nutricode 1** (Short stature/Stunting + Thinness/wasting)	48.4 (30)	51.9 (28)	27.1 (26)	15.6 (12)	50 (58)	22 (38)
**Nutricode 3** (Normal size + Thinness/wasting)	16.1 (10)	7.4 (4)	19.8 (19)	19.5 (15)	12.1 (14)	19.7 (34)
**Nutricode 4** (Short stature/Stunting + Normal weight)	21.0 (13)	24.1 (13)	29.2 (28)	42.9 (33)	22.4 (26)	35.3 (61)
**Nutricode 6** (Normal stature + Healthy weight)	12.9 (8)	14.8 (8)	21.9 (21)	22.1 (17)	13.8 (16)	22.0 (38)
**Nutricode 7** (Short stature/Stunting + Overweight/Obese)	1.6 (1)	1.9 (1)	2.1 (2)	0.0 (0)	1.7 (2)	1.2 (2)

N: total number in each group; %: percentage of total of each group; n: number of cases in each group.

**Table 4:** Relationship between the Nutrimetry (Nutricodes) and anaemia.

Nutrimetry					
	**Nutricode 1** (Short stature/Stunting + Thinness/Wasting)	**Nutricode 3** (Normal size + Thinness/Wasting)	**Nutricode 4** (Short stature/Stunting + Normal weight)	**Nutricode 6** (Normal size + Healthy weight)	**Nutricode 7** (Short stature/Stunting + Overweight/Obese)
	(N = 96)	(N = 48)	(N = 87)	(N = 54)	(N = 4)
**Anaemia**	% (n)	% (n)	% (n)	% (n)	% (n)
Yes	41.3 (69)	17.4 (29)	22.8 (38)	17.4 (29)	1.2 (2)
No	22.1 (27)	15.6 (19)	40.2 (49)	20.5 (25)	1.6 (2)

N: number of total cases of each nutricode; %: percentage of cases; n: number of cases of each nutricode with presence or absence of anaemia.

**Table 5:** Prevalence of the analysed intestinal parasitic infections stratified by municipality.

	All	Antsoamadiro	Fianaratsoa	Toliara
	(N = 289)	(N = 71)	(N = 59)	(N= 159)
	% (n)	% (n)	% (n)	% (n)
**Intestinal Parasites (IPs)**	91.0 (263)	85.9 (61)	84.7 (50)	95.6 (152)
*G. duodenalis*	82.0 (237)	67.6 (48)	79.7 (47)	89.3 (142)
*T. trichiura*	28.4 (82)	36.6 (26)	16.9 (10)	28.9 (46)
*A. lumbricoides*	21.5 (62)	31.0 (22)	13.6 (8)	20.1 (32)
*S. mansoni*	9.7 (28)	14.1 (10)	30.5 (18)	0.0 (00)
Coinfection 2 IPs	34.6 (100)	36.6 (26)	30.5 (18)	35.2 (56)
Coinfection ≥ 3 IPs	5.5 (16)	9.9 (7)	5.1 (3)	3.8 (6)

N: number of the total population and of each studied zone; %: percentage of cases; n: number of positive cases.

**Table 6:** Relationship between intestinal parasitic infections and indicators of nutritional status and anaemia.

	IPs (N = 263)	Co-Infection: *T. trichiura* + *G. duodenalis* (N = 69)	STH (N = 127)	*T. trichiura* (N = 82)	*A. lumbricoides* (N = 62)	*S. mansoni* (N = 28)	*G. duodenalis* (N = 237)
	% (n)	% (n)	% (n)	% (n)	% (n)	% (n)	% (n)
**Z < −2**	**HAZ**						
Short stature/Stunting							
Yes	65.4 (172)	73.9 (51)	79.5 (101)	78.0 (64)	87.1 (54)	78.6 (22)	63.7 (151)
No	34.6 (91)	26.1 (18)	20.5 (26)	22.0 (18)	12.9 (8)	21.4 (6)	36.3 (86)
**Z < −2**	**BMIZ**						
Thinness/Wasting							
Yes	46.0 (121)	76.8 (53)	70.1 (89)	72.0 (59)	72.6 (45)	53.6 (15)	52.3 (124)
No	54.0 (142)	23.2 (16)	29.9 (38)	28.0(23)	27.4 (17)	46.4 (13)	47.7 (113)
***Nutricode 1***	***Nutrimetry***						
(Short stature/Stunting + Thinness/wasting)							
Yes	64.6 (170)	55.1 (38)	55.1 (70)	53.7(44)	66.1 (41)	42.9 (12)	34.6 (82)
No	35.4 (93)	44.9 (31)	44.9 (57)	46.3 (38)	33.9 (21)	57.1 (16)	65.4 (155)
	**Anaemia**						
Yes	59.7 (157)	72.5 (50)	62.2 (79)	70.7 (58)	51.6 (32)	46.4 (15)	59.9 (142)
No	40.3 (106)	27.5 (19)	37.8 (48)	29.3 (24)	48.4 (30)	53.6 (13)	40.1 (95)

IPs: Intestinal parasite; STHs: Soil-transmitted helminths; N: total number of cases; %: prevalence over positive cases; n: number of positive cases with and without undernourishment and with and without anaemia.

## Data Availability

The data presented in this study are available on request from the corresponding author. The data are not publicly available due to privacy.

## Abbreviations

The following abbreviations are used in this manuscript:

BMIZ	Body mass index-for-age z-scores
bp	Base pair
CI	Confidence interval
epg	Eggs per gram
HAZ	Height-for-age z-scores
Hb	Haemoglobin
IDA	Iron deficiency anaemia
IPs	Intestinal parasites
IPIs	Intestinal parasitic infections
MPI	Multidimensional Poverty Index
NGO	Non-governmental organization
OR	Odds ratios
qPCR	Quantitative PCR
SF	Solvent-free
*ssu*	Small subunit ribosomal
STHs	Soil-transmitted helminths
WASH	Water, sanitation, and hygiene
WHO	World Health Organization
